# Expression and functions of adenylyl cyclases in the CNS

**DOI:** 10.1186/s12987-022-00322-2

**Published:** 2022-03-20

**Authors:** Karan Devasani, Yao Yao

**Affiliations:** Department of Molecular Pharmacology and Physiology, Morsani College of Medicine, University of South Florida, 12901 Bruce B. Downs Blvd., MDC 8, Tampa, FL 33612 USA

**Keywords:** Adenylyl cyclases, Blood–brain barrier, CNS, G-protein coupled receptors

## Abstract

Adenylyl cyclases (ADCYs), by generating second messenger cAMP, play important roles in various cellular processes. Their expression, regulation and functions in the CNS, however, remain largely unknown. In this review, we first introduce the classification and structure of ADCYs, followed by a discussion of the regulation of mammalian ADCYs (ADCY1-10). Next, the expression and function of each mammalian ADCY isoform are summarized in a region/cell-specific manner. Furthermore, the effects of GPCR-ADCY signaling on blood–brain barrier (BBB) integrity are reviewed. Last, current challenges and future directions are discussed. We aim to provide a succinct review on ADCYs to foster new research in the future.

## Background

One important mechanism that cells use to sense their environment is via receptor-mediated signaling. Specifically, environmental signals, such as chemokines and neurotransmitters, bind to receptors at plasma membrane and activate key intracellular signaling molecules (e.g., second messengers), transferring information from outside to inside. One ubiquitous second messenger in various cell types is cyclic adenosine 3′,5′-monophosphate (cAMP), which can act via either a kinase-dependent manner to induce protein phosphorylation or a kinase-independent manner to induce protein–protein interactions [1]. cAMP plays a pivotal role in a variety of fundamental cellular processes [2], and thus its level needs to be tightly regulated. Adenylyl cyclases (ADCYs) catalyze the production of cAMP from ATP, while phosphodiesterases (PDEs) degrade cAMP to 5′-AMP [3, 4]. This review summarizes the structure, regulation, expression and functions of ADCYs in the CNS.

## Classification of ADCYs

ADCYs are grouped into six different classes (class I-VI) based on their structural and domain organizations [5]. Class I is the gamma-proteobacterial type found mainly in gram-negative bacteria, such as *Escherichia coli* [6, 7]. Class II exists in pathogens that secrete toxin proteins, including *Bordetella pertussis* and *Bacillus anthracis* [8]. Class III is the universal or ancestral class of ADCYs found in both bacteria and eukaryotes. Since many of the class III ADCYs have been identified in higher eukaryotes and most thoroughly studied in mammals, they are also known as mammalian ADCYs. Class IV has been identified in *Yersinia pestis* and in ruminal bacteria *Aeromonas hydrophila* [5]. Class V and class VI are found in anaerobic bacterium *Prevotella ruminicola* and nitrogen fixing bacterium *Rhizobium etli*, respectively [9, 10]. The last two classes of ADCYs have not yet been structurally characterized. In this review, we focus on class III/mammalian ADCYs.

## Mammalian ADCYs

**Fig. 1:**
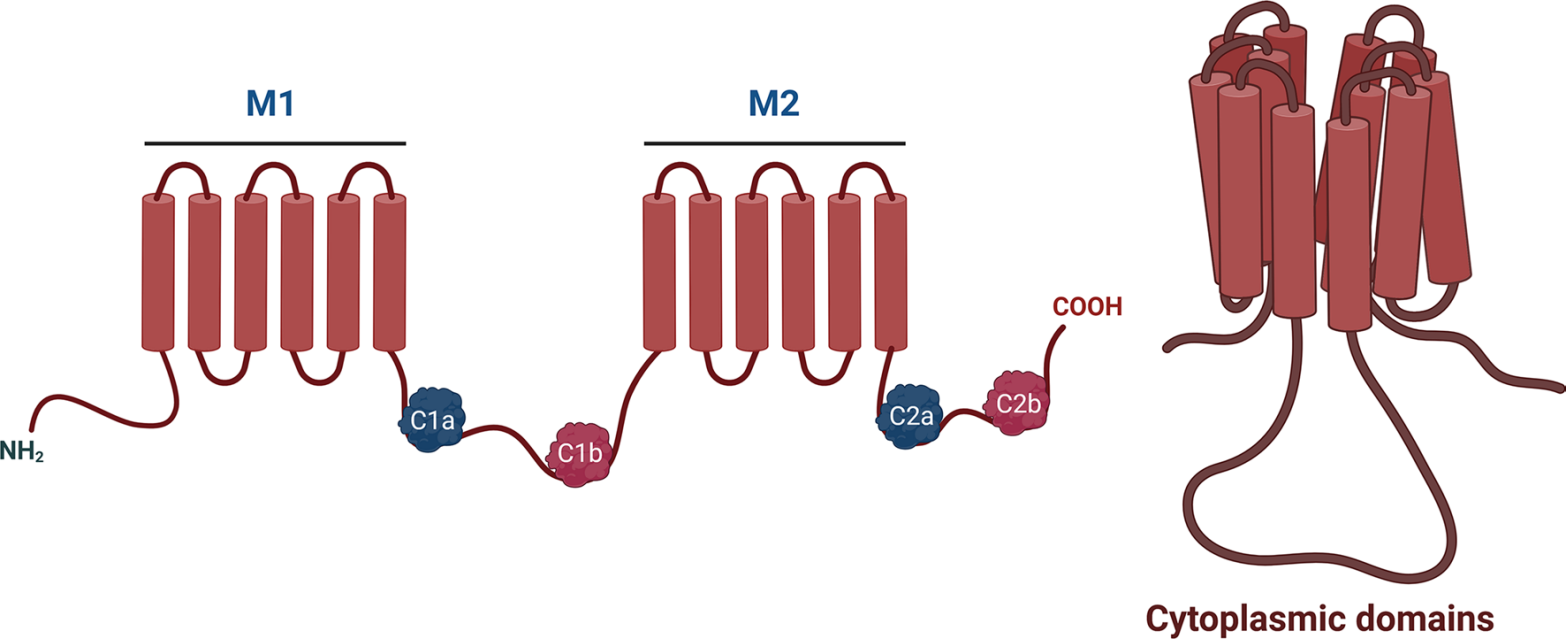
Structural illustration of transmembrane ADCYs. M1/M2: membrane-spanning domains; Catalytic site: C1a and C2a (navy blue); Regulatory site: C1b and C2b (red). Created with BioRender.com

Mammalian ADCYs have ten isoforms: nine transmembrane ADCYs (ADCY1-9) and one soluble ADCY (sADCY/ADCY10). All transmembrane ADCYs have a similar structure, but are different in their length and sequence at amino acids 1080–1353 [11] (Table 1). They consist of two discrete membrane-spanning (M1 and M2) domains with each containing six transmembrane alpha-helices, a single N-terminal cytosolic domain, and two cytoplasmic (C1 and C2) domains (Fig. 1). The C1 domain lies between two transmembrane domains, while the C2 domain is at the large C-terminus of the protein. These C1/C2 domains are subdivided into C1a/C2a and C1b/C2b subdomains (Fig. 1). The C1a and C2a subdomains are the catalytic site and highly conserved: they are structurally identical and homologous among all 9 transmembrane isoforms, while the C1b and C2b subdomains are the regulatory site [11, 12]. Unlike ADCY1-9, ADCY10 does not have clearly defined transmembrane domains (Table 1) [13]. Its catalytic domain is more related to bicarbonate-sensing ADCY from cyanobacteria than that of ADCY1-9.

**Table 1:** Chromosomal location, structure and length of mammalian ADCYs

Gene name	Chromosome (human)	Chromosome (mouse)	Structure	Length (human)	Length (mouse)
ADCY1	7p12	11A2		1119	1118
ADCY2	5p15	13C1		1091	1090
ADCY3	2p22-24	12A-B		1144	1145
ADCY4	14q11.2	14D3		1077	1077
ADCY5	3q13.2-q21	16B5		1261	1262
ADCY6	12q12-13	15F		1168	1165
ADCY7	16q12-13	8C3-D		1080	1099
ADCY8	8q24	15		1251	1249
ADCY9	16p13.3	16B1		1353	1353
ADCY10	1q24.2	1		1610	1614

Genetic studies have shown that *ADCY* genes are not clustered in the genome: each isoform is coded by a gene localized on a different chromosome [14, 15] (Table 1). This enables isoform-specific regulation of ADCYs.

## Regulation of ADCYs

**Fig. 2:**
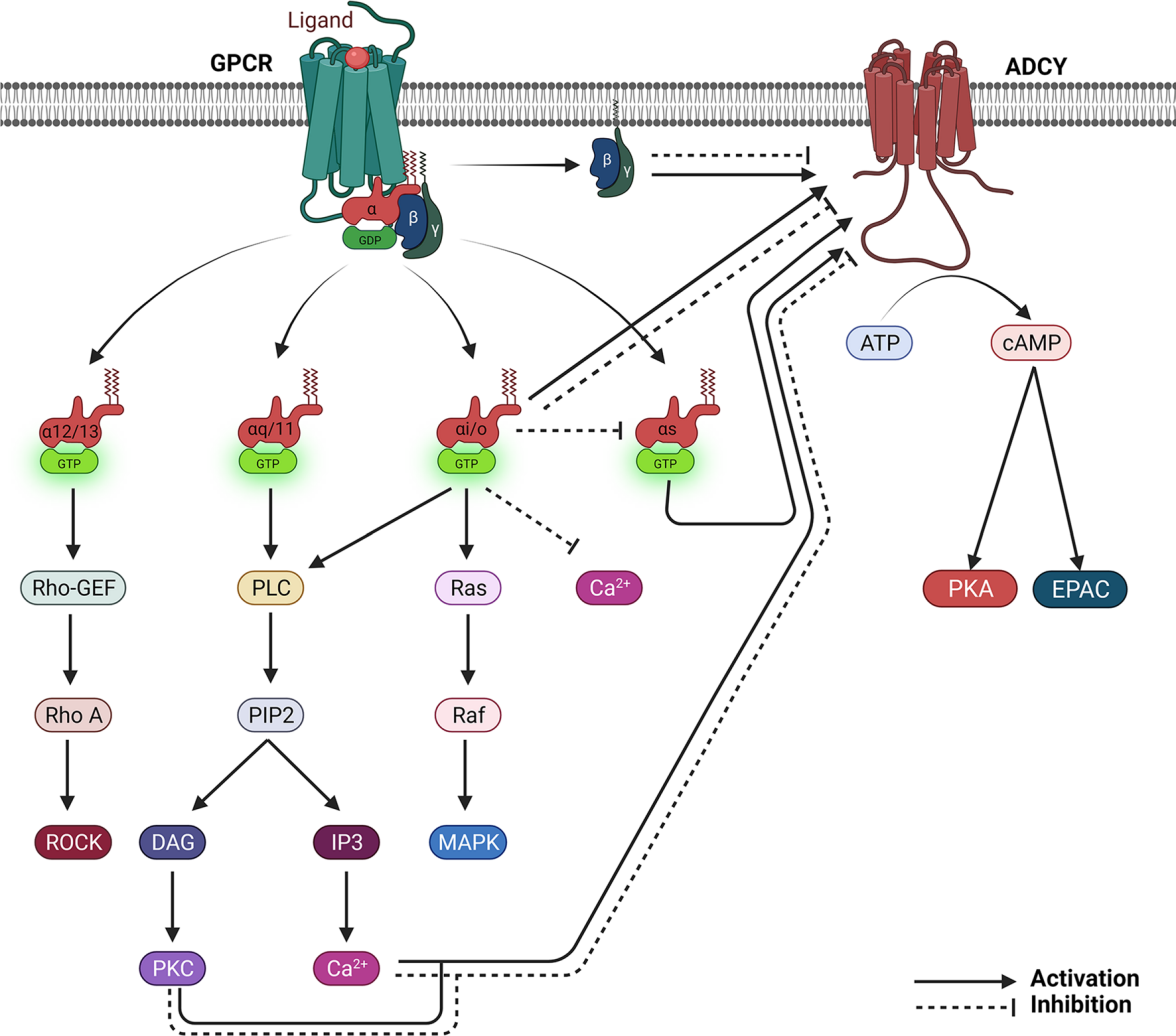
Schematic illustration of GPCR-ADCY signaling pathways. Upon ligand binding to the GPCR, GDP on G_α_ subunit is replaced with GTP, triggering the dissociation of G_βγ_ from G_α_. The dissociated G_α_ and G_βγ_ subunits interact with different effectors and signaling molecules. G_αs_ directly activates ADCY activity, converting ATP to cAMP, which activates PKA and EPAC. G_αi/o_ directly activates or inhibits ADCY activity depending on ADCY isoforms. In addition, G_αi/o_ can inhibit the stimulation of G_αs_ and Ca^2+^; and activate MAPK pathway and PLC. G_αq/11_ indirectly participates in the regulation of ADCY via Ca^2+^ and PKC, which are generated via PLC-DAG/IP3 signaling pathway. PKC and Ca^2+^ can either activate or inhibit ADCY activity in an isoform-specific manner. G_α12/13_ activates GEFs-RhoA signaling and does not seem to regulate ADCY activity. G_βγ_ activates or inhibits ADCY activity depending on its G_α_ partner and ADCY isoforms. Created with BioRender.com

ADCY activity is mainly regulated by G protein-coupled receptors (GPCRs). G protein is a heterotrimer containing α, β and γ subunits. Based on its function, G_α_ subunit is divided into four major categories: G_αs_, G_αi/o_, G_αq/11_ and G_α12/13_ (Fig. 2). Currently, there are five β and 11 γ subunits, which through forming highly active βγ heterodimers participate in the regulation of various biological processes [16]. Upon ligand binding, GPCRs change their confirmation replace GDP with GTP on G_α_ subunit, leading to dissociation of G_βγ_ complex. Then G_α_ and G_βγ_ independently activate downstream signaling cascades. The system returns to the resting state when ligands are released from GPCRs, which causes hydrolysis of GTP to GDP on G_α_ subunit and subsequent reassociation of G_βγ_ with G_α_ to form heterotrimers.

The effect of GPCRs on ADCY activity is dependent on the type of G protein (Fig. 2). Specifically, G_αs_ directly activates ADCYs, increasing cAMP production. G_αi/o_ directly activate or inhibit ADCY activity in an ADCY isoform-specific manner. G_αq/11_ indirectly regulates ADCY activity via protein kinase C (PKC) and/or Ca^2+^, which can activate or inhibit ADCY activity. G_α12/13_ does not seem to be involved in the regulation of ADCY activity. The dissociated G_βγ_ complex can either activate or inhibit ADCY activity depending on its G_α_ partner and ADCY isoforms. It should be noted that G_βγ_ can also be generated via modulatory protein GoLoco without activation of GPCRs. Similarly, G_βγ_ generated this way may have stimulatory or inhibitory effect on ADCY activity.

**Fig. 3:**
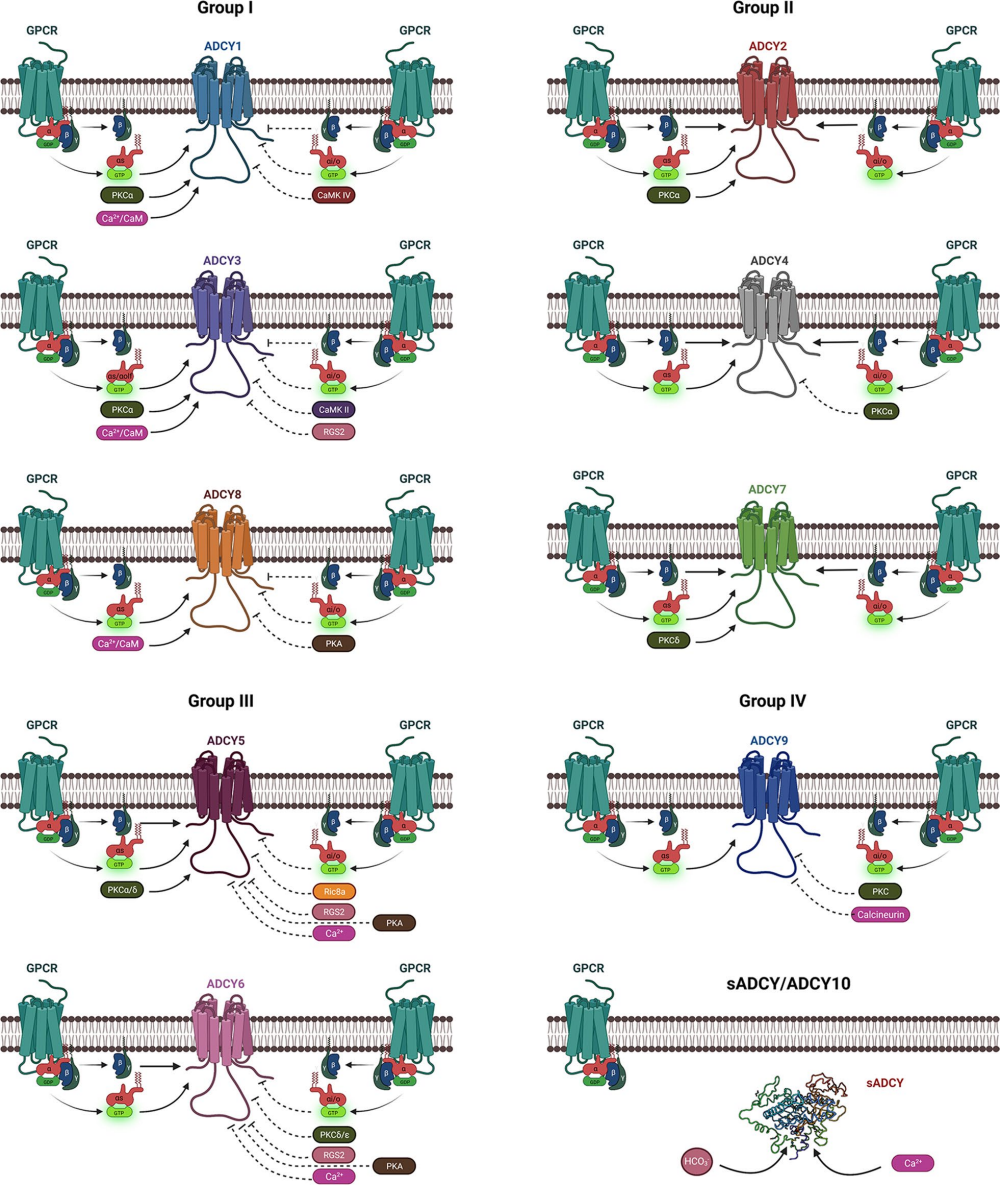
Diagram illustration of the regulation of mammalian ADCYs. Key regulators of mammalian ADCYs are summarized based on their groups. Unique regulators for each ADCY isoform are also illustrated. Created with BioRender.com

Based on their signaling properties, transmembrane ADCYs are further divided into four groups (Group I-IV). Group I is composed of ADCY1, ADCY3 and ADCY8; Group II includes ADCY2, ADCY4 and ADCY7; Group III consists of ADCY5 and ADCY6; and Group IV contains ADCY9 only. The unique features of these four groups of transmembrane ADCYs and soluble ADCY are discussed below and summarized in Fig. 3.

### Group I

Group I ADCYs are activated by G_αs_ and Ca^2+^/calmodulin, and inhibited by G_αi/o_ and G_βγ_. It should be noted that these ADCYs have different sensitivity to the stimuli. For example, ADCY3 and ADCY8 are five-fold less sensitive to Ca^2+^ than ADCY1 [17]. In addition, the activity of group I ADCYs can also be regulated by phosphorylation. For instance, PKCα activates ADCY1 and ADCY3; whereas calcium/calmodulin-dependent protein kinase IV (CaMKIV) inactivates ADCY1, CaMKII and regulator of G protein signaling 2 (RGS2) inactivate ADCY3, and PKA inactivates ADCY8 [18].

### Group II

Group II ADCYs are activated by both G_αs_ and G_βγ_ with a higher potency on G_αs_, but insensitive to Ca^2+^/calmodulin. Although group II ADCYs are not inhibited by G_αi/o_, it is assumed that G_βγ_ released from G_αi/o_ stimulation can synergistically stimulate them [11, 15, 19]. In addition, PKC regulates group II ADCYs in an isoform-specific manner. Specifically, PKCα activates ADCY2 but inactivates ADCY4, and PKCδ activates ADCY7 [18].

### Group III

Group III ADCYs are activated by G_αs_ and G_βγ_, but inhibited by G_αi/o_ and free Ca^2+^. Although most ADCYs are inhibited by high (non-physiological) concentration of Ca^2+^, ADCY5 and ADCY6 are inhibited by Ca^2+^ at sub-micromolar level [17], which may have important physiological implications. In addition, ADCY5 is also activated by PKCα/δ and inhibited by RGS2, PKA and Rica8; whereas ADCY6 is inhibited by RGS2, PKA and PKCδ/ε [18].

### Group IV

Group IV ADCY is activated solely by G_αs_. Unlike other transmembrane ADCYs, ADCY9 is insensitive to forskolin due to the lack of a key leucine residue in the catalytic cleft [20, 21]. Although ADCY9 is not regulated by G_αi/o_ or G_βγ_, calcineurin and PKC are able to inhibit its activity [18].

### sADCY/ADCY10

Unlike transmembrane ADCYs, ADCY10 is not associated with the membrane: it is diffusely distributed in the cytoplasm and nucleus [22]. Although ADCY10 is not regulated by G protein and insensitive to forskolin, it is activated by Ca^2+^ and bicarbonates [22, 23]. The negative regulators of ADCY10 have yet to be identified and the functions of ADCY10 remain largely unknown. It has been reported that nuclear ADCY10 is involved in gene regulation [24], while mitochondrial ADCY10 moderates oxidative phosphorylation in response to CO_2_/HCO_3_^−^ generated by citric acid cycle [25, 26].

## Expression and functions of ADCYs in the CNS

ADCYs are found in almost all cells and different cell types express distinct ADCY isoforms. The expression patterns of ADCY isoforms are mainly obtained from RNA-sequencing analyses (at the mRNA level). Currently, ADCY expression profiles at the protein level are limited, possibly due to the lack of isoform-specific antibodies.

ADCYs play a variety of important functions in the CNS, ranging from learning/memory to movements. Abnormal ADCY expression is found in and associated with many neurological disorders, including Alzheimer’s disease and depressive disorders. For example, loss of ADCY1 leads to impaired synaptic plasticity and deficits in spatial learning [27], while overexpression of ADCY1 in the forebrain enhances recognition and memory [28]. ADCY8 exerts similar functions as ADCY1 [29–35] and is associated with bipolar disorder [36] and post-traumatic stress disorder [37] in humans. ADCY3 is involved in olfactory-dependent learning and associated with major depressive disorder in humans [38, 39]. Loss of ADCY5 results in Parkinson-like motor dysfunction and locomotor impairment [40]. ADCY7 is linked to familial major depression in both mice and humans [41, 42]. It should be noted that not all ADCYs are well studied. The functions of ADCY2, ADCY4, ADCY6, ADCY9 and ADCY10 in the CNS remain largely unknown. Here, we discuss and summarize the expression (Table 2) and function (Table 3) of each ADCY isoform in the CNS.

**Table 2:** Region/cell-specific expression of ADCYs in the CNS

Isoforms	Sites of expression	Cell types
ADCY1	Piriform cortex^#^, CA1-CA2 of hippocampus^#^, dentate gyrus^#^, striatum^#^, amygdala^^^, thalamus^#^, and cerebellum^#^, cerebral cortex^^^, and olfactory bulb layers^*^	Neurons^#^, oligodendrocytes^^^, microglia^^^, astrocytes^^^, fibroblasts^*^ and endothelial cells^*^
ADCY2	Piriform cortex^#^, CA1 of hippocampus^#^, dentate gyrus^#^, striatum^#^, CA2-CA3 of hippocampus^^^, thalamus^^^, hypothalamus^^^, olfactory bulb layers^^^, cerebral cortex^^^, amygdala^*^, and cerebellum^*^	Neurons^#^, astrocytes^#^, fibroblasts^#^, smooth muscle cells^#^, oligodendrocytes^^^, microglia^*^, endothelial cells^*^, pericytes^*^
ADCY3	CA1-CA3 of hippocampus^#^, dentate gyrus^#^, cerebral cortex^^^, striatum^^^, amygdala^^^, thalamus^^^, hypothalamus^^^, cerebellum^^^, olfactory bulb layers^*^, piriform cortex^*^, and choroid plexus	Neurons^#^, astrocytes^#^, fibroblasts^#^, pericytes^#^, oligodendrocytes^^^, smooth muscle cells^*^, microglia^*^, endothelial cells^*^, and choroid plexus epithelial cells
ADCY4	Hippocampus^*^ and olfactory epithelium^*^	Endothelial cells^#^, neurons^*^, astrocytes^*^, fibroblasts^*^, smooth muscle cells^*^, oligodendrocytes^*^, microglia^*^, and pericytes^*^
ADCY5	Striatum^#^, hypothalamus^^^, olfactory bulb layers^^^, cerebral cortex^*^, piriform cortex^*^, amygdala^*^, CA1-CA3 of hippocampus^*^, dentate gyrus^*^, and thalamus^*^	Neurons^#^, oligodendrocytes^#^, fibroblasts^#^, smooth muscle cells^#^, astrocytes^*^, microglia^*^, endothelial cells^*^, and pericytes^*^
ADCY6	Piriform cortex^#^, amygdala^#^, CA1-CA3 of hippocampus^#^, dentate gyrus^#^, hypothalamus^#^, cerebellum^#^, choroid plexus^#^, olfactory bulb layers^^^, cerebral cortex^^^, striatum^^^, and thalamus^^^	Neurons^#^, astrocytes^#^, fibroblasts^#^, smooth muscle cells^#^, oligodendrocytes^#^, endothelial cells^#^, pericytes^#^, and microglia^*^
ADCY7	Thalamus^^^, and hypothalamus^^^, cerebral cortex^*^, amygdala^*^, corpus callosum^*^, cerebellum^*^, and olfactory system^*^	Fibroblasts^#^, smooth muscle cells^#^, microglia^#^, neurons^*^, astrocytes^*^, oligodendrocytes^*^, endothelial cells^*^, and pericytes^*^
ADCY8	Piriform cortex^#^, CA1-CA2 of hippocampus^#^, dentate gyrus^#^, thalamus^#^, hypothalamus^#^, cerebellum^#^, olfactory bulb^#^, cerebral cortex^*^, and amygdala^*^	Neurons^#^, astrocytes^#^, fibroblasts^*^, smooth muscle cells^*^, oligodendrocytes^*^, microglia^*^, and endothelial cells^*^
ADCY9	Olfactory bulb^#^, cerebral cortex^#^, piriform cortex^#^, CA1-CA3 of hippocampus^#^, dentate gyrus^#^, cerebellum^#^, amygdala^^^, thalamus^*^, and hypothalamus^*^	Neurons^#^, astrocytes^#^, fibroblasts^#^, smooth muscle cells^#^, oligodendrocytes^#^, microglia^#^, endothelial cells^#^, and pericytes^#^
ADCY10	Visual cortex^*^, hippocampus^*^, and cerebellum^*^, and choroid plexus	Neurons^*^, astrocytes^*^, fibroblasts^*^, smooth muscle cells^*^, oligodendrocytes^*^, microglia^*^, endothelial cells^*^, pericytes^*^, and choroid plexus epithelial cells

^#^High expression, ^^^Medium expression, ^*^Low expression

**Table 3:** ADCY functions and their associated diseases

Isoforms	Knockout/knockdown	Overexpression	Potential functions	Associated diseases	References
ADCY1	Yes^a^	Yes	Learning, memory, LTP, synaptic plasticity, drug dependency, nociception, and pain	Anxiety-like behavior, hearing impairment, sleep deprivation, schizophrenia, bipolar disorder, and autism	[28, 50, 53, 55–57]
ADCY2	–	–	Synaptic plasticity and neuropsychiatric functions	Bipolar disorder, anxiety, stress-like disorders, Lesch-Nyhan disease, and schizophrenia	[63–66]
ADCY3	Yes^a,b^	–	Odorant signaling, learning, and memory	Obesity, depression, and inflammatory bowel disease	[19, 38, 68, 70–72, 74–79, 190]
ADCY4	Yes^b^	–	–	–	[81]
ADCY5	Yes^a,c^	Yes	Learning, memory, synaptic plasticity, and extrapyramidal motor functions	Familial dyskinesia and facial myokymia, anxiety, depressive-like disorder, and movement disorders	[83–85, 89, 92–95]
ADCY6	Yes^a^	Yes	Metabolic functions and fluid homeostasis in kidney	Axoglial diseases and lethal congenital contracture syndrome	[97, 98, 100, 101]
ADCY7	Yes^c^	Yes	Alcohol dependency	Depression-like disorder, inflammatory bowel disease, Crohn’s disease, ulcerative colitis, and autoimmune diseases	[42, 108, 109]
ADCY8	Yes^a^	Yes	Learning, memory, LTP, synaptic plasticity, nociception, and pain	Dissociative amnesia, post-traumatic stress disorder, depression, and bipolar disorder	[29, 33, 37, 52, 112, 113]
ADCY9	Yes^a^	Yes	Learning and memory, immunological functions, and cardioprotective	Asthma, mood disorders, and bipolar disorder	[116, 119–121]
ADCY10	Yes^a^	–	Synaptic plasticity, learning and memory, ocular dominance plasticity, and fertilization	Infertility and absorptive hypercalciuria	[126, 132, 133]

^a^Global knockout, ^b^Conditional knockout, ^c^Knockdown

### ADCY1

#### Expression

*ADCY1* mRNA is transiently expressed in trigeminal nerve nuclei, striatum, dorsal thalamus, hippocampal interneurons, retinal ganglion cells and cerebellar Purkinje cells in early postnatal life [43]. In adulthood, however, its expression is confined to olfactory bulb, pineal gland, cortex, dentate gyrus, various thalamic nuclei, CA1 region of hippocampus and granule cells of the cerebellum [43, 44]. Bulk RNA-sequencing analysis showed that *ADCY1* expression was high in neurons and moderate in oligodendrocytes, microglia and astrocytes [45]. Single-cell RNA-sequencing study demonstrated high and low levels of *ADCY1* in vascular fibroblast-like cells and endothelial cells, respectively [46].

#### Function

As a Ca^2+^/calmodulin-sensitive ADCY, ADCY1 exerts important functions in neuronal development, a process that is critically regulated by calcium [47]. Correlation studies showed that hippocampal expression of ADCY1 was reduced during aging and increased during the acquisition of spatial learning [48, 49], highlighting a possible role of ADCY1 in learning/memory. In addition, ADCY1-null mice exhibited reduced long-term potentiation (LTP) in hippocampal mossy fibers, impaired cerebellar LTP, and higher threshold to inflammatory and chronic pain [50–52]. Consistent with these findings, overexpression of ADCY1 in forebrain led to elevated LTP, improved memory and decreased social ability via increased extracellular signal-related kinase (ERK1/2) [28]. These results suggest that ADCY1 regulates learning/memory, LTP and nociception.

Interestingly, FMR1-null mice, a rodent model of fragile X syndrome, exhibited increased ADCY1 expression and ADCY1-overexpression-like phenotype, including enhanced autism-related behaviors and increased ERK1/2 activity [53]. Loss of ADCY1 in FMR1-null background, however, reversed these changes [53]. These findings suggest that FMR1 inhibits ADCY1 expression, and that loss of FMR1-dependent suppression of ADCY1 is a cause for eccentric neuronal signaling in fragile X syndrome.

In humans, it has been reported that a nonsense mutation in *ADCY1* gene causes hearing impairment, deafness and loss of hair cell function [54, 55]. In addition, genome wide analysis studies (GWAS) showed that *ADCY1* polymorphism was associated with sleep deprivation, schizophrenia and bipolar disorder [56, 57].

### ADCY2

#### Expression

*ADCY2* mRNA is highly expressed in piriform cortex, hippocampus, dentate gyrus, striatum and thalamus [58]. Bulk RNA-sequencing study found that *ADCY2* was mainly expressed in astrocytes, neurons and oligodendrocytes precursor cells (OPCs); and to a lesser extent in oligodendrocytes and microglia [45]. Single-cell RNA-sequencing analysis showed that *ADCY2* was expressed at high levels in astrocytes, vascular fibroblast-like cells and smooth muscle cells, and at low levels in endothelial cells and pericytes [46]. At the protein level, ADCY2 expression has been found in mouse hippocampus [59], indicating a possible role in synaptic plasticity.

#### Function

The function of ADCY2 remains largely unknown. A correlation study found that ADCY2 was down-regulated throughout the hippocampus during the acquisition of spatial learning in mice [49], suggesting that ADCY2 may be involved in spatial learning and memory. In addition, it has been reported that P19 cells (embryonic carcinoma cells) up-regulate *ADCY2* during neuronal [60] and mesodermal [61] differentiation, highlighting a possible role of ADCY2 in cell differentiation during development.

A recent GWAS study revealed that *ADCY2* polymorphism was associated with neuropsychiatric disorders, including bipolar disorder [62, 63], anxiety and stress-like disorders [64], Lesch-Nyhan disease and schizophrenia [65, 66].

### ADCY3

#### Expression

*ADCY3* mRNA is highly expressed in olfactory sensory neurons (OSN), neuronal primary cilia, and dorsal root ganglion [67, 68]. Bulk and single-cell RNA-sequencing studies showed that *ADCY3* was highly expressed in neurons, OPCs, astrocytes, pericytes and vascular fibroblast-like cells; and moderately expressed in oligodendrocytes, microglia, endothelial cells and smooth muscle cells [45, 46]. At the protein level, ADCY3 is mainly found in primary cilia on choroid plexus cells and astrocytes [69].

#### Function

The high expression of ADCY3 in olfactory sensory neurons suggests that it may regulate odor/pheromone detection [67, 68]. Consistent with these results, ablation of ADCY3 leads to defective olfactory sensory neuron maturation and abnormal olfactory-based behavioral responses, including lack of preference for the test odorants in both sand-buried food task and odor-associated passive avoidance learning paradigm, absence of inter-male aggressiveness and male sexual behavior, and defective maternal behaviors [19, 70–72]. In addition, ADCY3-null mice also exhibit impaired learning/memory and short-term memory loss [38], highlighting an essential role of ADCY3 in learning/memory. Furthermore, dysregulation of ADCY3-mediated cAMP signaling in choroid plexus epithelial cells has also been suggested to contribute to the onset of hydrocephalus [73].

GWAS studies demonstrated that *ADCY3* polymorphism was associated with obesity [74–76], depression [77], and inflammatory bowel disease [78, 79].

### ADCY4

#### Expression

*ADCY4* mRNA is expressed at extremely low levels in various brain regions, including olfactory bulbs, cerebral cortex, hippocampus, amygdala, basal ganglia, thalamus, hypothalamus, pons, medulla and cerebellum [58, 80]. Bulk and single-cell RNA-sequencing analyses demonstrated that ADCY4 expression was predominantly detected in endothelial cells [45, 46]. One study reported ADCY4 expression in dentate gyrus and hippocampal CA1/CA3 regions at the protein level [59].

#### Function

The expression of *ADCY4* in hippocampus and dentate gyrus suggests a possible role in synaptic plasticity [59]. Although *ADCY4* is also detected in olfactory cilia [19], it does not seem to play a role in olfactory perception since it cannot rescue anosmia in ADCY3-null mice [19].

Outside the CNS, ADCY4 is mainly expressed in the kidney. However, loss of ADCY4 in kidney collecting duct principal cells fails to affect vasopressin-stimulated cAMP generation or sodium/water reabsorption [81], highlighting a dispensable role of ADCY4 in these cells.

### ADCY5

#### Expression

*ADCY5* mRNA is highly expressed in the olfactory system, piriform cortex and striatum; and weakly expressed in thalamus and hippocampus [58, 80]. Bulk and single-cell RNA-sequencing analyses revealed high levels of *ADCY5* in neurons, vascular fibroblast-like cells, smooth muscle cells, OPCs and oligodendrocytes; and low levels of *ADCY5* in microglia, astrocytes, pericytes and endothelial cells [45, 46]. Consistent with these findings, *ADCY5* mRNA is detected in cholinergic interneurons and GABAergic medium spiny neurons in the striatum [82, 83].

#### Function

In vitro study showed that P19 cells up-regulated *ADCY5* during neuronal differentiation [60], highlighting an important role of ADCY5 in neuronal maturation/function. Echoed with this result, knockdown of ADCY5 in nucleus accumbens decreases cAMP, leading to blood–brain barrier (BBB) disruption, social stress and depression-like behaviors [84]. Similarly, ADCY5-null mice exhibit poor stress-coping responses [85], indicating a critical role of ADCY5 in the regulation of anxiety and stress. In addition, loss of ADCY5 also impairs striatum-dependent learning, corticostriatal plasticity, dopamine signaling and motor activity [83, 86].

Outside the CNS, ADCY5 participates in the regulation of heart function. In vitro study showed that ADCY5 expression correlated with the appearance of beating cardiomyocytes and transcription of MLC1A (myosin light chain 1 atrial isoform) during mesodermal differentiation of P19 cells, highlighting an important role of ADCY5 in early cardiogenesis and cardiomyocyte differentiation [61]. In addition, deletion of ADCY5 improves basal left ventricular function [87, 88], protects the heart against chronic βAR stimulation [89] and age-related cardiomyopathy [90, 91]. These results indicate a detrimental role of ADCY5 in heart function.

*ADCY5* polymorphism has been linked to neuropsychiatric disorders. For example, a missense mutation (A726T) has been associated with familial dyskinesia with facial myokymia (FDFM) [92]. In addition, a homozygous missense or heterozygous de novo mutation (p.R418W) results in early onset of motor disability and movement disorder with severe intellectual disability [93–95].

### ADCY6

#### Expression

*ADCY6* has a similar but broader and higher expression pattern as *ADCY5*. In addition to the olfactory system, piriform cortex and striatum, *ADCY6* mRNA is also highly expressed in the limbic areas, including amygdala, hippocampus, dentate gyrus and hypothalamus [58, 80]. Bulk and single-cell RNA-sequencing analyses showed that ADCY6 was highly expressed in neurons, OPCs, oligodendrocytes, astrocytes, endothelial cells, pericytes and smooth muscle cells [45, 46].

#### Function

The function of ADCY6 in the CNS remains unknown. Outside the CNS, ADCY6 is involved in the pathogenesis of cardiac and renal disorders. In vitro study showed that P19 cells up-regulated *ADCY6* during mesodermal differentiation [61]. Expression of ADCY6 in the left ventricle of pigs with congestive heart failure increases cardiac contractility and ameliorates cardiac failure [96]. Although loss of ADCY6 does not affect basal cAMP level, it greatly reduces βAR-stimulated cAMP production [97, 98]. ADCY6-null mice display increased urine output, decreased urine osmolarity, reduced responsiveness to arginine vasopressin (AVP), and mild Bartter syndrome-like phenotype [99]. Together, these findings highlight important roles of ADCY6 in cardiac and renal functions. In addition, homozygous missense mutation (R1116C) in ADCY6 reduces myelination in peripheral nervous system, contributing to human axoglial diseases [100] and lethal congenital contracture syndrome [101]. ADCY6 has also been identified as a prognostic factor involved in DNA methylation-regulated immune processes in luminal-like breast cancer [102].

### ADCY7

#### Expression

*ADCY7* mRNA expression is restricted to thalamus and hypothalamus, with lower expression in cerebral cortex, amygdala, corpus callosum, cerebellum and olfactory bulbs [41, 58, 80]. Bulk and single-cell RNA-sequencing analyses showed that ADCY7 was highly expressed in microglia and vascular fibroblast-like cells [45, 46]. At the protein level, ADCY7 expression is mainly found in hippocampus, cerebellum, caudate-putamen, cerebral cortex and nucleus accumbens [103].

#### Function

Ethanol-induced GABAergic transmission in central amygdala neurons was ablated in ADCY7^+/^ brain slices [104], while mutant mice overexpressing human ADCY7 in the brain displayed higher plasma adrenocorticotropin and corticosterone levels after ethanol injection  [105]. These findings suggest that ADCY7 plays an important role in ethanol modulation of presynaptic GABA release, which may underlie ethanol-related behaviors such as anxiety and dependence.

There is also evidence suggesting that ADCY7 is involved in mood regulation and major depressive disorder. It has been reported that overexpression of ADCY7 in female mice increases depression-like behaviors, while ADCY7^+/−^ mice display decreased depression-like symptoms [42]. Consistent with this finding, a tetranucleotide repeat [(AACA)_7_] polymorphism in *ADCY7* is associated with depressive disorders in humans [42, 65]. Postmortem study found increased ADCY7 expression in the amygdala and anterior cingulate cortex of patients with depression [41].

In addition, ADCY7 is a major contributor of cAMP in T and B lymphocytes. Loss of ADCY7 leads to fewer leukocytes and higher mortality upon bacterial infections [106, 107], indicating an essential role of ADCY7 in immune responses. Consistent with these results, *ADCY7* polymorphism is associated with inflammatory bowel disease, Crohn’s disease, ulcerative colitis and autoimmune diseases [108, 109].

### ADCY8

#### Expression

During early postnatal life, *ADCY8* mRNA is expressed in hippocampal CA1 region, cortex, cerebellum, olfactory bulb, hypothalamus, amygdala and basal ganglia. In adulthood, *ADCY8* is found in olfactory bulb, cerebellum, hypothalamus, thalamus, hippocampal CA1 region, habenula, cerebral and piriform cortices [43, 110]. Bulk and single-cell RNA-sequencing analyses showed that ADCY8 was predominantly expressed in neurons, OPCs and astrocytes [45, 46].

#### Function

In vitro study showed that P19 cells up-regulated *ADCY8* during neuronal differentiation [60], suggesting a possible role of ADCY8 in neuronal development. In vivo study demonstrated that knockdown of ADCY8 ablated the midline-crossing of retinal neurons in zebrafish, resulting in mis-projections of exons to the ipsilateral tectum [111], highlighting an essential role of ADCY8 in axonal pathfinding. ADCY8-null mice showed defective short-term plasticity, impaired presynaptic/postsynaptic LTP and abnormal anxiety-like behaviors under stress [29, 33, 51]. In addition, ADCY8-null mice exhibited no reduction in allodynia and slightly reduced behavioral nociceptive responses to subcutaneous formalin injection or nerve injury [52]. ADCY1-null and ADCY1/ADCY8 double knockout mice, on the other hand, displayed more dramatic changes in these tests [52]. These findings indicate a relatively less important role of ADCY8 in behavioral responses to inflammation or nerve injury compared to ADCY1.

GWAS studies showed that *ADCY8* polymorphism was associated with various neuropsychiatric disorders, including dissociative amnesia, post-traumatic stress disorder, depression and bipolar disorder [37, 112, 113].

### ADCY9

#### Expression

*ADCY9* mRNA is broadly expressed in the brain with high levels in olfactory system, neocortex, piriform cortex, hippocampus, dentate gyrus, thalamus, hypothalamus and cerebellum [114, 115] Bulk and single-cell RNA-sequencing analyses showed that *ADCY9* was highly expressed in almost all cell types in the CNS, including neurons, OPC, oligodendrocytes, astrocytes, microglia, endothelial cells, pericytes, smooth muscle cells and vascular fibroblast-like cells [45, 46].

#### Function

Although ADCY9 is abundantly expressed in the brain, its function in the CNS remains largely unknown. Loss of ADCY9 leads to grade 1 ventricular diastolic dysfunction and embryonic lethality [116], preventing investigation of its function in adulthood. A study found reduced expression of *ADCY9* in the hippocampus in aged mice [48]. More importantly, *ADCY9* was significantly increased in mouse hippocampus after spatial learning and its expression correlated with animal performance in the Morris water maze test [48]. These findings suggest that ADCY9 may regulate cognitive function and learning/memory.

In addition, there is also evidence showing that ADCY9 modulates immune function. For example, it has been reported that ADCY9 regulates the chemotaxis of neutrophils and monocytes [117] as well as T cell function [11, 118].

GWAS studies found that ADCY9 polymorphism was associated with asthma [119, 120], mood disorders [121], and the efficacy of dalcetrapib, an antiatherogenic drug [122].

### ADCY10

#### Expression

Bulk RNA-sequencings analysis showed minimal expression of ADCY10 in neurons, OPCs, oligodendrocytes, astrocytes, microglia and endothelial cells [45]. Single-cell RNA-sequencing study found relatively high expression of ADCY10 in endothelial cells and astrocytes [46]. At the protein level, ADCY10 is found in astrocytes [123], developing neurons [124], and neurons of visual cortex, hippocampus and cerebellum [125, 126]. In addition, ADCY10 expression has also been found in the choroid plexus at both mRNA [127] and protein [128, 129] levels.

#### Function

ADCY10 activation in astrocytes increases cAMP level, induces glycogenolysis/glycolysis, and provides energy substrate for astrocytes and neurons [123], suggesting an important role in astrocyte-neuron metabolic coupling. Overexpression of ADCY10 in retinal ganglion and dorsal root ganglion cells promotes axonal outgrowth and growth cone elaboration, whereas inhibition of ADCY10 reverses these changes [124, 125], strongly indicating an essential role of ADCY10 in axonal outgrowth. Based on that ADCY10 is expressed in the choroid plexus and CO_2_ metabolism is linked to cerebrospinal fluid secretion [130], it is hypothesized that ADCY10 regulates cerebrospinal fluid homeostasis. This is evidence showing that increased ADCY10 expression caused by chloral hydrate-induced removal of cilia enhances transcytosis in choroid plexus epithelial cells [131]. Two ADCY10 knockout mouse lines have been generated: C1KO and C2KO, which prevent the expression of C1 and C2 domains, respectively. Both display defective sperm motility due to decreased cAMP production in testis and spermatozoa [126, 132], highlighting a crucial role of ADCY10 in male infertility.

A clinical study revealed that *ADCY10* polymorphism is associated with absorptive hypercalciuria and low spinal bone density [133].

## Effects of GPCR-ADCY signaling in BBB integrity

The BBB is a unique feature of CNS blood vessels. It is mainly composed of brain endothelial cells, pericytes, astrocytes, microglia, neurons and a non-cellular component—the basal lamina. By tightly regulating what enters/exits the CNS, the BBB maintains brain homeostasis [134, 135].

The effects of GPCR-ADCY signaling in BBB maintenance remain largely unclear, partially due to the complexity of GPCR-ADCY system. There are 10 different ADCY isoforms, which are coupled to distinct GPCRs in different cell types. However, there is evidence suggesting that certain GPCRs may regulate BBB integrity via ADCY activity, although the specific ADCY isoforms involved in each case remain unknown. Below we briefly discuss a few such GPCRs, including sphingosine 1-phosphate receptors (S1PRs), lysophosphatidic acid receptors (LPARs), cannabinoid receptors (CBs), adenosine receptors (ARs), G protein-coupled estrogen receptor 1 (GPER-1), complement C5a receptor (C5aR), somatostatin receptors (SSTRs), glucagon-like peptide-1 receptor (GLP1R), and hydrocarboxylic acid receptor 1 (HCAR1). The expression, G protein subtypes, and functions (in BBB integrity) of these GPCRs are summarized in Table 4.

**Table 4:** GPCR and ADCY expression and function on BBB integrity

GPCRs	Types of G proteins	BBB integrity	Cell types	References
S1PR1	Gα_i/o_	Increase	Astrocytes and endothelial cells	[136, 138–140]
S1PR2	Gα_i/o_, Gα_q/11_, and Gα_12/13_	Decrease	Pericytes, glia, fibroblasts, and endothelial cells	
S1PR3	Gα_i/o_, Gα_q/11_, and Gα_12/13_	Decrease	Astrocytes and endothelial cells	
S1PR5	G_αi/o_ and Gα_12/13_	Increase	Oligodendrocytes and endothelial cells	
LAPR1	Gα_i/o_, Gα_q/11_, and Gα_12/13_	Decrease	Microglia, oligodendrocytes, astrocytes, and endothelial cells	[147–150, 153, 155]
LAPR2	Gα_i/o_, Gα_q/11_, and Gα_12/13_	Decrease	Neuron, fetal astrocytes, and endothelial cells	
LAPR3	Gα_i/o_ and G_αq/11_	Decrease	Microglia, astrocytes, and endothelial cells	
LAPR6	G_αs_ and Gα_12/13_	Decrease	Microglia, oligodendrocytes, and endothelial cells	
CB1	G_αs_, Gα_i/_o, and G_αq_	Increase	Microglia and neurons	[156–160]
CB2	G_αs_, Gα_i/o_, and G_αq_	Increase	Microglia and neurons	
AR-A1	Gα_i/o_	Decrease	Microglia, neurons, oligodendrocytes, astrocytes, and endothelial cells	[139, 162, 163]
AR-A2A	G_αs_	Decrease	Microglia, neurons, and astrocytes	
GPER-1	G_αs_ and G_βγ_	Increase	Neurons	[166–168]
C5aR	Gα_i/o_	Decrease	Microglia, astrocytes, and neurons	[169, 170]
SSTRs	Gα_i/o_ and G_βγ_	Increase	Neurons	[172–174]
GLP1R	G_αs_	Increase	Microglia, astrocytes, neurons, and endothelial cells	[175–177]
HCAR1	Gα_i/o_	Increase	Astrocytes, neurons, and endothelial cells	[178–180]

### S1PRs

S1PRs are the receptors for sphingosine 1-phosphate, a signaling sphingolipid with a diverse range of functions. There are 5 subtypes of S1PRs (S1PR1-5), among which four (S1PR1-3 and S1PR5) have been shown to regulate BBB integrity. In the CNS, S1PR1 and S1PR3 are mainly expressed in astrocytes and endothelial cells; S1PR2 is found in pericytes, glial cells, endothelial cells and fibroblasts [136]; and S1PR5 is mainly found in oligodendrocytes and endothelial cells [137, 138]. S1PR1 is coupled to G_αi/o_; S1PR2 and S1PR3 are coupled to G_αi/o,_ Gα_q/11_ and Gα_12/13_; and S1PR5 is coupled to G_αi/o_ and Gα_12/13_ [139, 140]. Functional studies suggest that S1PR1 regulates BBB integrity. It has been reported that S1PR1/5 agonist siponimod (BAF-312) enhances BBB integrity and increases tight junction protein expression in an in vitro BBB model [141]. Consistent with this finding, S1PR1 functional antagonist (FTY720P) and endothelium-specific knockout of S1PR1 substantially increase BBB permeability to small tracers [142]. There is also evidence showing that S1PR1 can be targeted to facilitate CNS drug delivery. It has been shown that targeting S1PR with S1P and S1PR agonist fingolimod improves CNS drug delivery by reducing basal activity of P-glycoprotein (P-gp), an ATP-driven drug efflux pump, at the BBB and blood–spinal cord barrier, which significantly increases the uptake of radiolabeled P-gp substrates such as verapamil (three-fold), loperamide (five-fold) and paclitaxel (five-fold) [143, 144]. Similarly, pharmacological studies suggest that S1PR2 and S1PR3 function to decrease BBB integrity [145, 146]. S1PR2 antagonist ameliorates oxidative stress-induced cerebrovascular endothelial barrier impairment and reduces BBB leakage after ischemic injury in mice [145]. S1PR3 antagonist CAY10444 attenuates BBB damage by up-regulating tight junction proteins, reduces brain edema, and improves animal behavior in acute intracerebral hemorrhage [146]. S1PR5, on the other hand, seems to promote BBB integrity. It has bene reported that S1PR5-selective agonist improves BBB integrity in vitro and reduces trans-endothelial migration of monocytes. Echoed with these findings, knockdown of S1PR5 compromises BBB integrity and reduces the expression of tight junction proteins, P-gp and BCRP [138]. It should be noted, however, that the specific ADCY isoforms associated with S1PR1-3 and S1PR5 signaling remain unknown.

### LPARs

LPARs are the receptors for lysophosphatidic acid (LPA), a bioactive lipid with important functions in physiology and pathology. There are six subtypes of LPARs (LPAR1-6), among which four (LPAR1-3 and LPAR6) have been shown to regulate BBB integrity. LPAR1 is mainly expressed in astrocytes, microglia, oligodendrocytes and endothelial cells; LPAR2 in endothelial cells, neuron and fetal astrocytes; LPAR3 in microglia, astrocytes and endothelial cells; and LPAR6 in microglia, oligodendrocytes, endothelial cells [147, 148]. LPAR1-3 signal through Gα_i/o_, Gα_q/11_ and Gα_12/13_ [147, 149], while LPAR6 signals through Gα_s_ and Gα_12/13_ [149]. All of these LPARs (LPAR1-3 and LPAR6) function to decrease BBB integrity. It has been shown that LPA decreases tight junction protein expression and transendothelial electrical resistance via LPAR6 in rat brain endothelial cells [150]. Echoed with this finding, intravenous injection of LPA up-regulates LPAR1-3 expression and transiently increases BBB permeability [151]. Additionally, LPA and amitriptyline have been shown to reduce basal P-gp activity through LPAR1 signaling without affecting the activity of MRP2 (multidrug resistance-associated protein 2) or BCRP (breast cancer resistance protein) in both rat brain capillaries and a rat model of amyotrophic lateral sclerosis [152]. Similarly, LPAR ligand gintonin increases tight junction spaces and decreases tight junction protein expression in human brain microvascular endothelial cells. Gintonin has been shown to enter the brain via LPAR1/LPAR3 and enhance BBB permeability to various tracers in vivo [153]. Consistent with these findings, gintonin enhances CNS delivery of donepezil in a time-dependent manner via LPAR1/3 [154]. LPAR inhibitors (HA130, PF8380 and BrP-LPA), on the other hand, reverse BBB damage and enhance tight junction protein expression after ischemic stroke [155].

### CBs

CBs have two subtypes (CB1 and CB2), both of which mediate BBB regulation in healthy and injured/diseased conditions. In the CNS, CB1 and CB2 are mainly expressed in neurons and microglia [156, 157]. They can stimulate and/or inhibit various ADCY isoforms independently. It has been shown that activation of CB1 and CB2 by cannabinoid agonists stimulates group II ADCYs (ADCY2, ADCY4 and ADCY7) through G_αs_, but inhibits other transmembrane ADCYs (ADCY1, ADCY3, ADCY5, ADCY6, ADCY8 and ADCY9) through G_αi/o_ and G_αq_ [158, 159]. In vitro study showed that pharmacological activation of CB1 but not CB2 restored tight junction stability in HIV-1-induced BBB disruption model [160]. Consistent with this finding, CB1-specific cannabinoid agonists inhibited HIV-1 Gp120-mediated BBB damage and prevented down-regulation of tight junction proteins both in vitro and in vivo [160]. Interestingly, CB2-selective agonist O-1966 prevented LPS-induced loss of tight junction proteins in brain microvascular endothelial cells [157]. These findings suggest a protective role of CB1/2 in BBB integrity.

### ARs

ARs are the receptors for adenosine, a purine nucleoside released by neurons and glial cells. There are four subtypes of ARs (A1, A2A, A2B and A3), among which AR-A1 and AR-A2A are involved in BBB regulation. In the CNS, AR-A1 and AR-A2A are predominantly expressed in microglia, oligodendrocytes, astrocytes, neurons and endothelial cells [161]. AR-A1 inhibits ADCY activity through G_αi/o_, whereas AR-A2A stimulates ADCY activity via G_αs_ [139, 162]. Although coupled to different G proteins, both ARs function to compromise BBB integrity. It has been shown that activation of AR-A1 and AR-A2A increases BBB permeability and reduces tight junction protein expression [163]. In addition, AR agonists have been used to facilitate the entry of intravenously administered molecules into the brain [164]. For example, AR-A2A agonist lexiscan has been reported to inhibit the expression of P-gp and BCRP and increase the accumulation of the epirubicin, a P-gp substrate and chemotherapeutic drug, in mouse brain [165]. In addition, lexiscan has also been shown to increase paracellular leakage in cultured brain endothelial cells, enabling a wider therapeutic window for therapeutics to enter the brain [162].

### GPER-1

GPER-1, also known as GPR30, is a novel estrogen receptor highly expressed in neurons [166]. Activation of GPER-1 stimulates ADCY activity via G_αs_ and G_βγ_ [167]. GPER-1 activation has been shown to reduce BBB leakage and increase tight junction proteins after ischemic injury [168], highlighting a protective role in BBB integrity.

### C5aR

C5aR is the receptor for C5a, a potent proinflammatory peptide generated during complement system activation. In the CNS, C5aR is constitutively expressed in astrocytes, microglia and neurons. Activation of C5aR inhibits ADCY activity via G_αi/o_ [169, 170]. In vitro study showed that activation of C5aR increased BBB permeability and decreased tight junction protein expression [171], suggesting a detrimental role of C5aR in BBB integrity.

### SSTRs

SSTRs mediate the effect of somatostatin, a neuropeptide with important functions in modulating cortical circuits and cognition. There are five subtypes of SSTRs (SSTR1-5), all of which are mainly expressed in neurons and inhibit ADCY activity via Gα_i/o_ and G_βγ_ [172, 173]. In vitro study showed that somatostatin and selective SSTR agonists maintained BBB integrity and restored ZO-1 organization in cytokine- and LPS-treated human brain endothelial cells [174], suggesting that SSTR activation protects BBB integrity.

### GLP1R

GLP1R mediates the function of glucagon-like peptide-1, a short peptide hormone secreted by intestinal enteroendocrine L cells and certain neurons. In the CNS, GLP1R is mainly expressed in astrocytes, neurons, microglia and endothelial cells [175]. Activation of GLP1R stimulates ADCY activity via G_αs_ [176]. In vitro study showed that GLP-1 increased tight junction protein expression and decreased paracellular permeability in brain capillary endothelial cells via cAMP-PKA signaling pathway [177], indicating a protective role in BBB integrity.

### HCAR1

HCAR1, also known as GPR81, is expressed in endothelial cells, astrocytes and neurons [178, 179]. It inhibits ADCY activity through G_αi/o_ [178, 179]. In vitro study showed that LPS reduced the expression of HCAR1 and tight junction proteins and increased BBB permeability in rat brain microvascular endothelial cells [180]. In addition, activation of HCAR1 stimulates mitochondrial biogenesis and regulates monocarboxylate transporter expression in brain endothelial cells, which are crucial for the metabolism and function of the neurovascular unit [181, 182]. These results suggest a possible role of HCAR1 in BBB maintenance.

## Conclusions and future directions

Since the identification of cAMP as an important second messenger, substantial progress has been made with respect to the structure, expression, regulation and functions of ADCYs. There are, however, still several key questions that need to be answered in future research.

First, the expression profiles of ADCYs at the protein level remain largely unknown, possibly due to the lack of isoform-specific antibodies. Current knowledge on ADCY expression is mainly at the mRNA level. Future research should focus on addressing this bottleneck by developing isoform-specific antibodies and innovative genetic tools (e.g., reporter mouse lines).

Second, there is a lack of genetic knockout/overexpression models for certain ADCY isoforms. For example, the phenotypes of ADCY2, ADCY4 and ADCY7 global knockout mice as well as ADCY2, ADCY3, ADCY4 and ADCY10 overexpression mice remain unknown. In addition, the cell-specific conditional knockout mice for many ADCY isoforms are still lacking. Furthermore, there are currently few compound knockout mice available, which are useful in dissecting the roles of ADCYs with compensatory/overlapping functions. Generating these genetic tools will enable loss-of-function studies and substantially move the field forward.

Third, there is a lack of isoform-specific pharmacological reagents for ADCYs. Incomplete pharmacological characterization of mammalian ADCYs has resulted in misconceptions/errors in the selectivity of certain compounds [183]. The lack of selectivity and potency of pharmacological reagents has often resulted in inaccurate or even faulty conclusions in ADCY research. Future research should focus on screening and identifying isoform-specific ADCY activators and inhibitors.

Fourth, the association profiles between ADCYs and GPCRs in different cell types remain unknown. Establishing a cell-specific GPCR-ADCY association profile will significantly enrich our knowledge in GPCR-ADCY signaling. Together with the cell-specific expression profiles of GPCRs and ADCYs, this association profile makes it possible to determine the crosstalk between various signaling pathways, promoting more accurate and safer treatments.

Fifth, more efficient and specific detection approaches are needed for ADCY research. Although multiple methods exist to measure cAMP levels in cells, these approaches are mostly end-point assays and unable to reflect cAMP levels in real time or that generated by a specific ADCY isoform [184, 185]. Although fluorescence resonance energy transfer (FRET)-based cAMP biosensors allow the cAMP detection in living cells in real time [186, 187], they usually have low efficiency and sensitivity. More sensitive and rapid approaches are needed. In addition, biosensors that are able to target different subcellular compartments may help study localized cAMP dynamics [188, 189].

Last, the functions of ADCYs are not fully understood. For example, the roles of ADCYs in CNS barriers (e.g., BBB, blood-CSF barrier and brain-CSF barrier) and the underlying molecular mechanisms are only partially understood. In addition, the functions of ADCY isoforms in human diseases and the links between ADCY gene polymorphisms and human diseases remain largely unknown. With the generation of novel tools (isoform-specific antibodies and genetic mouse lines), we expect to determine the functional significance of each ADCY isoform in a cell-specific manner.

## Data Availability

Not applicable.

## Abbreviations

ADCYs: Adenylyl cyclases
ARs: Adenosine receptors
BBB: Blood–brain barrier
cAMP: Cyclic adenosine 3′,5′-monophosphate (cAMP)
CaMKII: Calcium/calmodulin-dependent protein kinase II
CaMKIV: Calcium/calmodulin-dependent protein kinase IV
C5aR: Complement C5a receptor
CBs: Cannabinoid receptors
GLP1R: Glucagon-like peptide-1 receptor
GPCRs: G protein-coupled receptors
GPER-1: G protein-coupled estrogen receptor 1
GWAS: Genome wide analysis studies
HCAR1: Hydrocarboxylic acid receptor 1
LTP: Long-term potentiation
LPARs: Lysophosphatidic acid receptors
OPC: Oligodendrocyte’s precursor cells
PKA: Protein kinase A
PKC: Protein kinase C
RGS2: Regulator of G protein signaling 2
SSTRs: Somatostatin receptors
S1PRs: Sphingosine 1-phosphate receptors
